# Improving physician documentation for malnutrition: A sustainable quality improvement initiative

**DOI:** 10.1371/journal.pone.0287124

**Published:** 2023-08-10

**Authors:** Brittany E. Levy, Jennifer T. Castle, Wesley S. Wilt, Kelly Fedder, Jeremy Riser, Erin D. Burke, Jon S. Hourigan, Avinash S. Bhakta

**Affiliations:** 1 Graduate Medical Education, General Surgery Residency Program, University of Kentucky College of Medicine, Lexington, Kentucky, United States of America; 2 Department of Clinical Nutrition, Center for Health Services Research, University of Kentucky College of Health Sciences, Lexington, Kentucky, United States of America; 3 Division of Colorectal Surgery, Department of Surgery, University of Kentucky College of Medicine, Lexington, Kentucky, United States of America; King Saud University College of Applied Medical Sciences, SAUDI ARABIA

## Abstract

This study compares documentation and reimbursement rates before and after provider education in nutritional status documentation. Our study aimed to evaluate accurate documentation of nutrition status between registered dietitian nutritionists and licensed independent practitioners before and after the implementation of a dietitian-led Nutrition-Focused Physical Exam intervention at an academic medical center in the southeastern US. ICD-10 codes identified patients from 10/1/2016-1/31/2018 with malnutrition. The percentage of patients with an appropriate diagnosis of malnutrition and reimbursement outcomes attributed to malnutrition documentation were calculated up to 24 months post-intervention. 528 patients were analyzed. Pre-intervention, 8.64% of patients had accurate documentation compared to 46.3% post-intervention. Post-intervention, 68 encounters coded for malnutrition resulted in an estimated $571,281 of additional reimbursement, sustained at 6, 12, 18, and 24 months. A multidisciplinary intervention improved physician documentation accuracy of malnutrition status and increased reimbursement rates.

## Introduction

Malnutrition, or more specifically undernutrition, occurs when good health is limited by adequate consumption of calories and essential nutrients [1, 2]. Multicenter and international studies estimate that 20–60% of hospitalized patients are malnourished [2–6]. Malnutrition is associated with poor clinical outcomes, such as impaired wound healing [7–9], a longer length of stay [10–12], morbidity, and increased mortality [13, 14]. Additionally, providing adequate care for malnourished patients requires resource-intensive interventions and specialized nutrition support professionals, both of which contribute to the cost of caring for these patients [15, 16].

Despite the increased costs associated with the provision of care, hospitals struggle to capture appropriate reimbursement for malnourished patients due to both under-documentation and improper documentation of a malnutrition diagnosis [17–19]. Surgical patients, especially oncologic patients, have an exceptionally high prevalence of malnutrition, upwards of 60% [20]. Unfortunately, when these patients’ nutrition status is not addressed by licensed independent practitioners (LIPs), their nutrition interventions can be delayed or withheld [21]. While proper documentation of malnutrition in hospitalized patients by LIPs has been shown to positively affect hospital reimbursement, many hospitals utilize registered dietician nutritionists (RDNs) to screen and document these findings [9, 22, 23]. Unfortunately, thorough documentation does not directly impact reimbursement, leading to potential undercharging for a patient’s medical complexity [23, 24]. In 2012, the Academy of Nutrition and Dietetics and the American Society for Parenteral and Enteral Nutrition developed a consensus statement on the appropriate criteria to accurately identify malnutrition in adults [2]. Methods to assess patients for malnutrition include the Subjective Global Assessment (SGA) scoring system and the Nutrition-Focused Physical Exam (NFPE), both of which have utility in clinical settings [25–27].

The Medicare Severity-Diagnostic Related Group (MS-DRG) classification established by the Centers for Medicare & Medicaid Services (CMS) is determined by the principal diagnosis. Hospital reimbursement rates are based on the average cost required to care for patients with diagnoses of that MS-DRG. This reimbursement value increases depending on major complications and comorbidities (MCCs) or complications and comorbidities (CCs) associated with the care of that patient. A secondary diagnosis of malnutrition, depending on its severity and corresponding International Classification of Diseases, 10^th^ revision code (ICD-10), can qualify as both an MCC or a CC and can thus increase reimbursement. Additionally, interdisciplinary communication between LIPs and RDNs can improve accurate reimbursement for malnourished hospitalized patients by improving accurate documentation [24, 28–31].

Studies have shown that the implementation of a structured assessment for nutrition status in hospitalized patients can improve the detection and documentation of malnutrition, with a resulting positive impact on reimbursement [32]. Most studies evaluating malnutrition diagnosing and hospital reimbursement employ National Risk Screening (NRS) or SGA screening systems for nutrition status assessment. Unfortunately, few studies following this design have been conducted in the US on surgical patients [29, 30, 31]. Our study aimed to evaluate accurate documentation of nutrition status between RDNs and LIPs before and after the implementation of a dietitian-led NFPE intervention at an academic medical center in the southeastern US.

## Methods

Due to the retrospective nature of this study, with no more than minimal harm to the study subjects, a waiver of informed consent was approved by our Institutional Review Board; IRB approval number 58780. Data were anonymized before analysis.

### Intervention

Malnutrition scoring systems such as the NFPE were introduced to providers in April 2017. Registered dieticians provided education to physicians and advanced practice providers regarding best practices for documentation and diagnosis of malnutrition. Didactic sessions included demonstrations on how to use the NFPE, interactive case presentations, and hands-on learning of where to appropriately document this diagnosis in the medical chart.

#### Study population

As part of a retrospective study, the electronic medical records of surgical patients from the University of Kentucky were queried from October 1, 2016, through January 31, 2018. Using ICD-10 codes related to malnutrition, patient malnutrition status of mild (E44.1), moderate (E44.0), severe (E43), and unspecified (E46) protein-calorie malnutrition were identified. All patients over the age of 18, admitted to a surgical service (vascular surgery, general surgery, colorectal surgery, surgical oncology) and evaluated by an RDN, were included in our analysis.

Patients were grouped based on their relationship to the intervention completed in April 2017. Therefore, patients from October 1, 2016, through March 31, 2017, were considered the pre-intervention cohort. Patients that were seen between April 1, 2017, and July 31, 2017, the three months following the intervention, were considered a transitional cohort. The transitional cohort was excluded from the final cost analysis. Finally, patients from August 1, 2017, through January 31, 2018, were considered the post-intervention patient cohort.

#### Outcome variables

Following the data query, patient medical records were analyzed to determine the congruency of the documentation between the registered dietician and physician diagnoses. Patient cohorts were analyzed for both pre-intervention and post-intervention, broken out by surgical service line.

Using Medicare and Medicaid weighted DRG multipliers, estimated reimbursement outcomes attributed to malnutrition documentation were calculated. Reimbursements were followed at 6 months, 12 months, 18 months, and 24 months post-intervention to identify the sustainability of the intervention.

#### Statistical analysis

Standard statistical analysis was applied to this dataset to provide the best depiction of data trends. Paired T-tests were utilized to compare pre-implementation and post-implementation averages. Additionally, non-linear generic method of moments (GMM) parameters were estimated for the study timeframe to determine the correlation between values. All statistics were done in Statistical Analysis System (SAS) statistical modeling software.

## Results

### Study population

In total, 528 patients were included for analysis. The pre-intervention cohort consisted of 194 patients, while the post-intervention group included 334 surgical patients. Transitional cohort patients were not included in the analysis. In both the pre-intervention and post-intervention groups, there were no statistical differences between demographics, length of stay, insurance status, age, cancer diagnosis, or ventilation rates (Table 1).

**Table 1:** Comparison of patient characteristics pre-intervention and post-intervention.

Variable	Period		p-value
	Pre-value (n = 194)	Post-value (n = 334)	
Length of stay, days	15.22	14.09	0.3743
Case mix indices	3.65	4.22	0.0873
Age	59.44	60.34	0.5237
Male	52.58% (102)	58.08% (194)	0.2375
White	96.91% (188)	95.81% (320)	0.6397
Appalachian	60.82% (118)	64.07% (214)	0.4567
Medicaid	24.23% (47)	25.45% (85)	0.8349
Ventilated	29.38% (57)	40.42% (135)	0.0114
ICU length of stay, days	3.86	4.36	0.4496
GI cancer diagnoses	11.34% (22)	8.98% (30)	0.4489

#### Diagnosis documentation correlations

**Fig 1:**
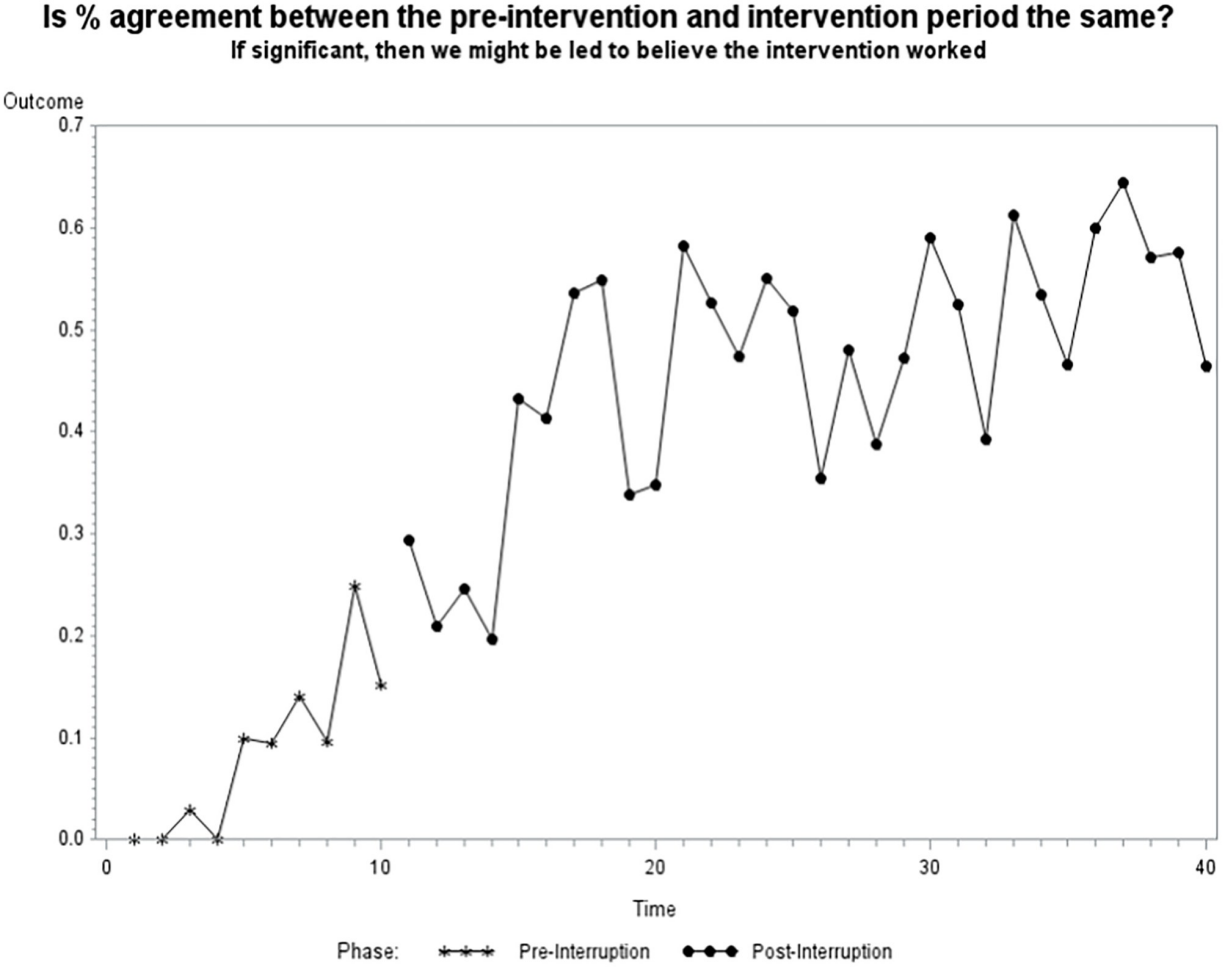
Estimated congruency between RDN and LIP documentation over the study period.

Before the educational, interdisciplinary intervention, 8.4% of patients had congruent documentation between dieticians and providers. Following the intervention, 46.31% of documentation was congruent (p<0.001) (Table 2). When comparing patient time cohorts, before the intervention there is a monthly increase of about 2.35% in agreement. Following the educational intervention, the correlation between RDN and LIP documentation was about 13%. The correlation slope began to decrease by 1.45% starting at 11 weeks post-intervention. Despite the decreasing slope, the positive correlation of 0.8% persists through 30 weeks post-intervention (Table 3). No post-intervention washout phase or drift-back to previous documentation incongruencies was seen in the 30 weeks post-intervention (Fig 1).

**Table 2:** Pre-intervention and post-intervention documentation congruency.

Pre-Intervention Mean	Post-Intervention Mean	Difference	95% CL		S.D.	p-value
			Lower	Upper		
8.64%	46.31%	37.67%	-0.4608	-0.2926	0.1138	<0.001

S.D. = standard deviation

**Table 3:** Nonlinear GMM correlation estimates.

Variable	Estimate	Approximate Standard Error	Approximate Pr > |t|
**Intercept**	-0.043	0.0085	< .0001
**Pre-intervention slope**	0.023	0.002	< .0001
**Level of change following the intervention**	0.132	0.048	0.009
**Difference in pre- & post- intervention trends**	-0.015	0.003	< .0001
**Post-intervention slope**	0.009	0.002	< .0001

#### Cost

Before the intervention period, 26 visits were estimated to have malnutrition diagnosis codes correctly applied to malnourished patients, resulting in a total of $278,566 additional reimbursement directly attributed to appropriate documentation of malnutrition based on Medicaid and Medicare reimbursement rates. During the transition phase, $204,594 of reimbursement related to 25 malnutrition-coded visits was estimated. Post-intervention, 68 visits were estimated to have a malnutrition diagnosis affected DRG, resulting in $571,281 of additional reimbursement. Follow-up estimates were completed at 6-month intervals through 24 months following the intervention. The estimated reimbursements related to malnutrition diagnosis codes rose from $669,129 at 6 months to $705,109, $704,102, and $835,634 at 12 months, 18 months, and 24 months, respectively (Table 4).

**Table 4:** Reimbursement related to documentation congruency.

**Pre-Period (Study)**		
**Payor Group**	**Estimated Number of Visits Where Malnutrition Dx Code Affected DRG Assignment (a)**	Estimated Additional Reimbursement Due to Malnutrition Dx Code (b*c)
Medicaid	8	$75,064
Medicare/Managed Care/Other	18	$203,501
**Total**	**26**	**$278,566**
**Transition Period (Study)**		
**Payor Group**	**Estimated Number of Visits Where Malnutrition Dx Code Affected DRG Assignment (a)**	Estimated Additional Reimbursement Due to Malnutrition Dx Code (b*c)
Medicaid	9	$56,527
Medicare/Managed Care/Other	16	$148,068
**Total**	**25**	**$204,594**
**Post Period (Study)**		
**Payor Group**	**Estimated Number of Visits Where Malnutrition Dx Code Affected DRG Assignment (a)**	Estimated Additional Reimbursement Due to Malnutrition Dx Code (b*c)
Medicaid	23	$198,277
Medicare/Managed Care/Other	45	$373,003
**Total**	**68**	**$571,281**
**Initial 6 Months Following Study**		
**Payor Group**	**Estimated Number of Visits Where Malnutrition Dx Code Affected DRG Assignment (a)**	Estimated Additional Reimbursement Due to Malnutrition Dx Code (b*c)
Medicaid	21	$183,947
Medicare/Managed Care/Other	48	$485,182
**Total**	**69**	**$669,129**

## Discussion

Malnutrition is associated with poor outcomes in surgical patients. While commonly documented by RDNs, there is frequently incongruent documentation by LIPs resulting in improper reimbursement for malnourished patients. Our study identified a successful quality improvement initiative led by RDNs to improve documentation of malnutrition by LIPs, which resulted in improved hospital reimbursement for malnourished patients.

Before our intervention, 8.4% of patients carried a billable diagnosis of malnutrition. This is similar to published rates of 0.6–18.0% based on population studies at discharge by Tobert et. al. [33], which identified a total of 5.8 million patient discharges over 2 years from 105 institutions with a reported malnutrition diagnosis rate of 4%. Hospital systems with improved diagnosis rates were associated with higher hospital rankings and improved patient satisfaction. The lack of LIP diagnosis, across specialties, raises concern regarding the identification of at-risk patients, given the known poor outcomes associated with malnourished patients [10]. Our study is the first to identify a sustainable intervention to improve the documentation and identification of malnourished surgical patients by LIPs. Following the intervention, 46.3% of patients had congruent documentation between the RDN diagnosis and LIP diagnosis.

Our intervention centered on an RDN-led educational campaign to educate LIPs on the utilization of the NFPE and associated diagnosis documentation. Studies have shown that the implementation of a structured assessment for nutrition status in hospitalized patients can improve the detection and documentation of malnutrition, with a resulting positive impact on reimbursement. In one such German study of 1244 patients undergoing elective surgery [34], the National Risk Screening (NRS) scoring system was used to screen patients’ nutritional status, and an appropriate ICD-10 code was assigned to each patient based on their NRS score. A simulation was run to assess the economic impact of proper coding based on the German-DRG system, and the authors found that the generated profit for each patient at risk for malnutrition was €263.96. In another similar German study of 541 patients in a gastroenterology ward, the SGA scoring system was used for screening and economic analysis of proper coding based on the ICD-10/German-DRG system which found a reimbursement increase of €360 per malnourished patient [35]. A Portuguese study of 129 patients in an internal medicine ward, using both the NRS and SGA scoring systems, also concluded that proper coding resulted in an overall increase in hospital reimbursement [36]. Other studies demonstrating shortfalls in hospital reimbursement due to poor documentation and coding of malnutrition also exist [17, 37]. Our study reveals a 3-times increase in reimbursement from the pre-intervention phase of $278,566 to the 24-month follow-up of $835,634 across all general surgery subspecialties.

Healthcare costs related to malnutrition are significant. Direct and indirect costs related to malnutrition are cited as up to 49 billion dollars; however, even these estimates are related to coded diagnoses rather than actual numbers of malnourished patients [38]. The cost of care for malnourished patients is increased with a longer length of hospital stay, surgical site infections, and in-hospital mortality [16, 29, 39]. In Canada, the Malnutrition Task Force utilized the SGA to identify sources of increased hospital costs in malnutrition. In 956 patients, hospital length of stay for both medical and surgical patients was increased by 23%, and 31%, respectively [16]. This corresponded to a 31% and 34% increased cost of hospital stay, respectively, when compared to well-nourished patients. Lee et. al. found surgical patients presenting with small bowel obstructions had a 4.2 times increased rate of in-hospital mortality if they also suffered severe protein-calorie malnutrition [21]. This study provides an intervention to improve the LIP identification of malnourished patients using the NFPE.

Physician awareness regarding malnutrition indicators and documentation is imperative to optimal patient care and appropriate reimbursement. Increased reimbursement funds could provide opportunities for funding pre-habilitation programs and other intensive nutrition programs to improve patient outcomes. While this study identifies a single application of multidisciplinary involvement to improve accurate documentation, hospital billing, and patient care, interdisciplinary collaboration benefits all vulnerable patient populations and should be pursued in all aspects of patient care. Additionally, similar multidisciplinary approaches for accurate documentation may aid in billing for other patient comorbidities, which may require significant healthcare resources.

### Limitations

The limitations of this study are related to the estimated reimbursement rates based on Medicare and Medicaid DRG data. Institutional cost data was not available for analysis due to the inability to identify patients who were not coded by a diagnosis code by LIPs in our financial system. However, Medicare and Medicaid DRG reimbursement is a widely accepted estimation of costs and hospital financial data. While clinical outcomes such as length of stay, adverse outcomes, and mortality were not assessed in this quality improvement project, further research is needed to determine if accurate documentation has a meaningful impact on these important clinical metrics.

## Conclusions

Overall, our study details the outcomes of an RDN-led educational quality improvement initiative to improve diagnosis ICD coding for malnourished patients. Our quality improvement initiative resulted in a sustainable 3-fold increase in reimbursement for malnourished patients and improved congruency between RDN and LIP documentation from 8.4% to 46.3%. Further quality improvement initiatives are needed to improve outcomes for malnourished patients and reduce overall hospital costs for this vulnerable population. Identification of malnourished patients is the first step to improving outcomes via targeted quality improvement initiatives. Therefore, further initiatives to improve interdisciplinary engagement should be explored to improve high-quality healthcare in surgical patients. Our project may inform other academic medical centers on how to proceed with quality improvement initiatives on similar patient outcomes topics.

## Supporting information

S1 Dataset (ZIP)

## References

[1] Maleta K. Undernutrition. Malawi Med J. 2006 Dec;18(4):189–205. . 27529011 PMC3345626

[2] White JV, Guenter P, Jensen G, Malone A, Schofield M; Academy Malnutrition Work Group; A.S.P.E.N. Malnutrition Task Force; A.S.P.E.N. Board of Directors. Consensus statement: Academy of Nutrition and Dietetics and American Society for Parenteral and Enteral Nutrition: characteristics recommended for the identification and documentation of adult malnutrition (undernutrition). JPEN J Parenter Enteral Nutr. 2012 May;36(3):275–83. doi: 10.1177/0148607112440285 22535923

[3] Naber TH, Schermer T, de Bree A, Nusteling K, Eggink L, Kruimel JW, et al. Prevalence of malnutrition in nonsurgical hospitalized patients and its association with disease complications. Am J Clin Nutr. 1997 Nov;66(5):1232–9. doi: 10.1093/ajcn/66.5.1232 9356543

[4] Pirlich M, Schütz T, Norman K, Gastell S, Lübke HJ, Bischoff SC, et al. The German hospital malnutrition study. Clin Nutr. 2006 Aug;25(4):563–72. doi: 10.1016/j.clnu.2006.03.005 16698132

[5] Lew CCH, Yandell R, Fraser RJL, Chua AP, Chong MFF, Miller M. Association between malnutrition and clinical outcomes in the intensive care unit: a systematic review. [Formula: see text] JPEN J Parenter Enteral Nutr. 2017 Jul;41(5):744–58. doi: 10.1177/0148607115625638 26838530

[6] Kang MC, Kim JH, Ryu SW, Moon JY, Park JH, Park JK, et al; Korean Society for Parenteral and Enteral Nutrition (KSPEN) Clinical Research Groups. Prevalence of malnutrition in hospitalized patients: a multi-center cross-sectional study. J Korean Med Sci. 2018 Jan 8;33(2):e10. doi: 10.3346/jkms.2018.33.e10 29215819 PMC5729651

[7] Winter TA, Lemmer ER, O’Keefe SJ, Ogden JM. The effect of severe undernutrition, and subsequent refeeding on digestive function in human patients. Eur J Gastroenterol Hepatol. 2000 Feb;12(2):191–6. doi: 10.1097/00042737-200012020-00010 10741934

[8] Mechanick JI. Practical aspects of nutritional support for wound-healing patients. Am J Surg. 2004 Jul;188(1A Suppl):52–6. doi: 10.1016/S0002-9610(03)00291-5 15223503

[9] Barker LA, Gout BS, Crowe TC. Hospital malnutrition: prevalence, identification, and impact on patients and the healthcare system. Int J Environ Res Public Health. 2011 Feb;8(2):514–27. doi: 10.3390/ijerph8020514 21556200 PMC3084475

[10] Lim SL, Ong KC, Chan YH, Loke WC, Ferguson M, Daniels L. Malnutrition and its impact on cost of hospitalization, length of stay, readmission and 3-year mortality. Clin Nutr. 2012 Jun;31(3):345–50. doi: 10.1016/j.clnu.2011.11.001 22122869

[11] Leiva Badosa E, Badia Tahull M, Virgili Casas N, Elguezabal Sangrador G, Faz Méndez C, Herrero Meseguer I, et al. Hospital malnutrition screening at admission: malnutrition increases mortality and length of stay. Nutr Hosp. 2017 Jul 28;34(4):907–13. doi: 10.20960/nh.657 29095016

[12] Treber LA, Harris MA. Effect of early nutrition intervention on patient length of stay. J Am Dietetic Assn. 1996 Sept;96(9)Suppl:A29. doi: 10.1016/S0002-8223(96)00407-5

[13] Kubrak C, Jensen L. Malnutrition in acute care patients: a narrative review. Int J Nurs Stud. 2007 Aug;44(6):1036–54. doi: 10.1016/j.ijnurstu.2006.07.015 16996065

[14] Holmes S. The effect of undernutrition in hospitalised patients. Nurs Stand. 2007 Nov 27-Dec 4;22(12):35–8. doi: 10.7748/ns2007.11.22.12.35.c6242 18087876

[15] Goates S, Du K, Braunschweig CA, Arensberg MB. Economic burden of disease-associated malnutrition at the state level. PLoS One. 2016 Sep 21;11(9):e0161833. doi: 10.1371/journal.pone.0161833 27655372 PMC5031313

[16] Curtis LJ, Bernier P, Jeejeebhoy K, Allard J, Duerksen D, Gramlich L, et al. Costs of hospital malnutrition. Clin Nutr. 2017 Oct;36(5):1391–6. doi: 10.1016/j.clnu.2016.09.009 27765524

[17] Kellett J, Kyle G, Itsiopoulos C, Naunton M, Luff N. Malnutrition: the importance of identification, documentation, and coding in the acute care setting. J Nutr Metabol. 2016;2016:9026098. doi: 10.1155/2016/9026098 27774317 PMC5059542

[18] Phillips W, Browning M. A clinician’s guide to defining, identifying and documenting malnutrition in hospitalized patients. Practical Gastroenterol. 2017 Nov;41(11):19–33.

[19] Lazarus C, Hamlyn J. Prevalence and documentation of malnutrition in hospitals: A case study in a large private hospital setting. Nutr Dietetics. 2005 Aug 15;62(1):41–7. doi: 10.1111/j.1747-0080.2005.tb00008.x

[20] Viana ECRM, Oliveira IDS, Rechinelli AB, Marques IL, Souza VF, Spexoto MCB, et al. Malnutrition and nutrition impact symptoms (NIS) in surgical patients with cancer. PLoS One. 2020 Dec 15;15(12):e0241305. doi: 10.1371/journal.pone.0241305 33320857 PMC7737886

[21] Lee MJ, Sayers AE, Drake TM, Singh P, Bradburn M, Wilson TR, et al; NASBO Steering Group and NASBO Collaborators. Malnutrition, nutritional interventions and clinical outcomes of patients with acute small bowel obstruction: results from a national, multicenter, prospective audit. BMJ Open. 2019 Jul 27;9(7):e029235. doi: 10.1136/bmjopen-2019-029235 31352419 PMC6661661

[22] Funk KL, Ayton CM. Improving malnutrition documentation enhances reimbursement. J Am Diet Assoc. 1995 Apr;95(4):468–75. doi: 10.1016/S0002-8223(95)00123-9 7699190

[23] Khan M, Hui K, McCauley SM. What is a registered dietitian nutritionist’s role in addressing malnutrition? J Acad Nutr Diet 2018;18(9):1804. doi: 10.1016/j.jand.2018.06.013 Available at: What Is a Registered Dietitian Nutritionist’s Role in Addressing Malnutrition? (jandonline.org) Accessed 04/25/2023.

[24] Phillips W. Coding for malnutrition in the adult patient: what the physician needs to know. Pract Gastroenter 2014 Sept;133:56–64. doi: 10.1002/ncp.10426

[25] Fontes D, Generoso SdeV, Toulson Davisson Correia MI. Subjective global assessment: a reliable nutritional assessment tool to predict outcomes in critically ill patients. Clin Nutr. 2014 Apr;33(2):291–5. doi: 10.1016/j.clnu.2013.05.004 23755841

[26] Detsky AS, McLaughlin JR, Baker JP, Johnston N, Whittaker S, Mendelson RA, et al. What is subjective global assessment of nutritional status? JPEN Parenter Enteral Nutr. 1987 Jan-Feb;11(1):8–13. doi: 10.1177/014860718701100108 3820522

[27] Phillips JW, Janowski M, Brennan H, Leger-LeBlanc G. [Abstract] Nutrition focused physical exam improves accuracy of malnutrition diagnosis. J Acad Nutr Diet. 2019 Sept;119(Suppl 2):568. doi: 10.1016/j.jand.2019.06.031

[28] Yinusa G, Scammell J, Murphy J, Ford G, Baron S. Multidisciplinary provision of food and nutritional care to hospitalized adult in-scoping review. J Multidiscip Healthc 2021 Feb 22;14:459–91. doi: 10.2147/JMDH.S255256 33654405 PMC7910096

[29] Yin L, Cheng N, Chen P, Zhang M, Li N, Lin X, et al. Association of malnutrition, as defined by the PG-SGA, ESPEN 2015, and GLIM criteria, with complications in esophageal cancer patients after esophagectomy. Front Nutr 2021 Apr 26;8:632546. doi: 10.3389/fnut.2021.632546 . 33981719 PMC8107390

[30] Almeida AI, Correia M, Camilo M, Ravasco P. Nutritional risk screening in surgery: valid, feasible, easy! Clin Nutr (Edinburgh, Scotland) 2012 Apr;31(2):206–11. doi: 10.1016/j.clnu.2011.10.003 22051119

[31] Håkonsen SJ, Pedersen PU. Bath-Hextall F, Kirkpatrick P. Diagnostic test accuracy of nutritional tools used to identify undernutrition in patients with colorectal cancer: a systematic review. JBI Database System Rev Implement Rep 2015 May 15;13(4);141–87. doi: 10.11124/jbisrir-2015-1673 26447079

[32] Mitchell LJ, Ball LE, Ross LJ, Barnes KA, Williams LT. Effectiveness of dietetic consultations in primary health care: a systemized review of randomized controlled trials. J Acad Nutr Diet. 2017 Dec;117(12):1941–62. doi: 10.1016/j.jand.2017.06.364 28826840

[33] Tobert CM, Mott SL, Nepple KG. Malnutrition diagnosis during adult inpatient hospitalizations: analysis of a multi-institutional collaborative database of academic medical centers. J Acad Nutr Diet. 2018 Jan;118(1):125–31. doi: 10.1016/j.jand.2016.12.019 28416434

[34] Thomas MN, Kufeldt J, Kisser U, Hornung HM, Hoffmann J, Andraschko M, et al. Effects of malnutrition on complication rates, length of hospital stay, and revenue in elective surgical patients in the G-DRG-system. Nutrition. 2016 Feb;32(2):249–54. doi: 10.1016/j.nut.2015.08.021 26688128

[35] Ockenga J, Freudenreich M, Zakonsky R, Norman K, Pirlich M, Lochs H. Nutritional assessment and management in hospitalized patients: implication for DRG-based reimbursement and health care quality. Clin Nutr. 2005 Dec;24(6):913–9. doi: 10.1016/j.clnu.2005.05.019 16046034

[36] Fernandes AC, Pessoa A, Vigario MA, Jager-Wittenaar H, Pinho J. Does malnutrition influence hospital reimbursement? A call for malnutrition diagnosis and coding. Nutrition. 2020 Jun;74:110750. doi: 10.1016/j.nut.2020.110750 32222583

[37] Gout BS, Barker LA, Crowe TC. Malnutrition identification, diagnosis and dietetic referrals: Are we doing a good enough job? Nutrition Dietetics. 2009 Dec 3;66(4):199–256. doi: 10.1111/J.1747-0080.2009.01372.X

[38] Doley J, Phillips W. Coding for malnutrition in the hospital: does it change reimbursement? Nutr Clin Pract. 2019; Dec 34(6):823–31. doi: 10.1002/ncp.10426 31650622

[39] Tsantes AG, Papadopoulos DV, Lytras T, Tsantes AE, Mavrogenis AF, Koulouvaris P, et al. Association of malnutrition with surgical site infection following spinal surgery: systematic review and meta-analysis. J Hosp Infect. 2020 Jan;104(1):111–9. doi: 10.1016/j.jhin.2019.09.015 31562915

